# Sex differences in biological aging and the association with clinical measures in older adults

**DOI:** 10.1007/s11357-023-00941-z

**Published:** 2023-09-25

**Authors:** Aung Zaw Zaw Phyo, Peter D. Fransquet, Jo Wrigglesworth, Robyn L. Woods, Sara E. Espinoza, Joanne Ryan

**Affiliations:** 1 Biological Neuropsychiatry & Dementia Unit, School of Public Health and Preventive Medicine, Monash University, 553, St. Kilda Road, Melbourne, VIC 3004 Australia; 2 School of Psychology, Deakin University, Burwood, Melbourne, VIC 3125 Australia; 3 ASPREE Research Unit, School of Public Health and Preventive Medicine, Monash University, Melbourne, VIC 3004 Australia; 4 Center for Translational Geroscience, Department of Medicine, Cedars-Sinai Medical Center, Los Angeles, CA USA

**Keywords:** Sex differences, Epigenetic aging, DNA methylation, Frailty, Brain-predicted age difference, Health markers

## Abstract

Females live longer than males, and there are sex disparities in physical health and disease incidence. However, sex differences in biological aging have not been consistently reported and may differ depending on the measure used. This study aimed to determine the correlations between epigenetic age acceleration (AA), and other markers of biological aging, separately in males and females. We additionally explored the extent to which these AA measures differed according to socioeconomic characteristics, clinical markers, and diseases. Epigenetic clocks (HorvathAge, HannumAge, PhenoAge, GrimAge, GrimAge2, and DunedinPACE) were estimated in blood from 560 relatively healthy Australians aged ≥ 70 years (females, 50.7%) enrolled in the ASPREE study. A system-wide deficit accumulation frailty index (FI) composed of 67 health-related measures was generated. Brain age and subsequently brain-predicted age difference (brain-PAD) were estimated from neuroimaging. Females had significantly reduced AA than males, but higher FI, and there was no difference in brain-PAD. FI had the strongest correlation with DunedinPACE (range *r*: 0.21 to 0.24 in both sexes). Brain-PAD was not correlated with any biological aging measures. Significant correlations between AA and sociodemographic characteristics and health markers were more commonly found in females (e.g., for DunedinPACE and systolic blood pressure *r* = 0.2, *p* < 0.001) than in males. GrimAA and Grim2AA were significantly associated with obesity and depression in females, while in males, hypertension, diabetes, and chronic kidney disease were associated with these clocks, as well as DunedinPACE. Our findings highlight the importance of considering sex differences when investigating the link between biological age and clinical measures.

**Supplementary Information:**

The online version contains supplementary material available at 10.1007/s11357-023-00941-z.

## Introduction

In 2020, approximately 10% of the world’s population was over 65 years of age [1]; and the proportion of older adults is growing at a faster rate than all other age groups. As aging represents the most profound risk factor for many chronic diseases, the prevalence of age-related diseases such as dementia and ischemic heart disease is also proportionately increasing [2]. The geroscience hypothesis posits that by targeting aging mechanisms, we can potentially reduce the risk of several age-related diseases and extend the health span (i.e., duration of life lived in good health) [3]. As such, increasing attention has been placed on the hallmarks of aging which may better capture underlying biological differences and thus an individual’s risk of disease.

One of the most studied and promising aging biomarkers is epigenetic age. It estimates biological age based on DNA methylation (DNAm) levels at multiple cytosine-phosphate-guanine dinucleotides (CpGs) across the genome [4–9]. While epigenetic age can be estimated from any tissue, when measured in the blood, it reflects system-wide biological age [4, 10]. Over the previous decade, a number of epigenetic clock algorithms have been developed [4–9]. The first-generation epigenetic clocks, namely, HorvathAge [5] and HannumAge [6], were generated to predict chronological age using the specific CpGs sites which are strongly associated with chronological age. HorvathAge [5] was developed on 353 CpGs sites across multiple tissue samples whereas HannumAge [6] was trained on 71 CpGs sites from blood samples. Second-generation clocks such as PhenoAge [7] and GrimAge [8, 11] were trained in blood samples by incorporating additional age-related clinical markers. PhenoAge [7] used 513 CpGs sites which are related to chronological age and nine phenotypic aging biomarkers including albumin, creatinine, and C-reactive protein levels whereas GrimAge [8] considered 1030 CpGs sites associated with smoking-pack-years and a selection of plasma proteins including adrenomedullin, beta-2-microglobulin, and cystatin-C. Further, using the same set of 1030 CpGs that underline the original GrimAge, GrimAge2 was recently developed by additionally incorporating two new DNAm-based plasma proteins—high sensitivity C-reactive protein, and hemoglobin A1C [11]. The second-generation clocks have been found to outperform the first-generation clocks in the risk prediction of age-related diseases and all-cause mortality [5–8, 11, 12]. For the first- and second-generation clocks, the residual taken from regressing epigenetic age on chronological age is referred to as epigenetic age acceleration (AA) [10]. A positive value of AA indicates an older epigenetic age relative to chronological age, which has been shown to be predictive of several age-related diseases [10]. More recently, a third generation, the Pace of Aging (DunedinPACE) was developed using 173 CpGs sites in blood samples [9]. In contrast to prior generations, DunedinPACE was built by incorporating longitudinal within-person physiological change together with methylation pattern and thus specifically designed to measure the rate of aging [9].

Other measures have also been developed to estimate biological aging in recent years. Another system-wide measure of biological aging is the deficit accumulation frailty index (FI), a multidimensional tool that measures the accumulation of health deficits across a range of physical, psychological, and mental processes [13]. Unlike AA, FI can capture the clinical state of age-related physiological decline in which a person is more susceptible to developing adverse health outcomes [14, 15]. There are also aging biomarkers that target specific organs. Brain age is estimated using neuroimaging data, and an older brain relative to chronological age, positive brain-predicted age difference (brain-PAD), indicates accelerated brain aging [16]. In recent years, evidence has found that accelerated brain aging was predictive of cognitive performance, and physical disability, particularly in older people [17–19]. In literature, few studies have investigated the associations between epigenetic clocks and frailty and/or brain age, showing inconsistent relationships between these biological aging measures [20–22]. Further, all these previous studies were investigated using only first or second generations of epigenetic clocks. Thus, it is yet unclear to what extent these other biological aging indices are strongly correlated with different generations of epigenetic AA, and thus, it remains unclear to what extent these capture different aspects of the aging process.

On average, females have a longer lifespan than males [23] but may carry a higher disease and disability burden [24]. Such discrepancies may reflect differences in biological phenotypes, genetic constitutions, and physiological traits between males and females [23, 25], In terms of social behaviors, there are recognized sex differences in health-related behaviors, and in healthcare seeking, including accessing social and familial support [26]. Given these disparities, it is important to understand how the relationships between AA and health status might differ between males and females.

This study aimed to determine the correlation between epigenetic aging, with other measures of biological aging (i.e., system-wide FI, and brain-PAD), in an initially healthy population of males and females aged ≥ 70 years. We additionally explored the extent to which these AA measures were associated with sociodemographic characteristics, clinical measures, and chronic conditions, separately in males and females.

## Methods

### Study population

This study sub-sampled 560 Australians from the ASPREE (ASPirin in Reducing Events in the Elderly) study, who provided blood samples at baseline [27]. Full details regarding the sampling procedure, inclusion and exclusion criteria, and study design of the ASPREE study have been published previously [27, 28]. Briefly, in Australia, participants aged ≥ 70 years were recruited through a partnership with general practices, from March 2010 through December 2014. The ASPREE exclusion criteria included a history of cardiovascular disease (CVD), atrial fibrillation, anemia, dementia, a score of < 78 on the Modified Mini-Mental State examination (3MS), uncontrolled high blood pressure (i.e., ≥ 180/ ≥ 105 mm Hg), or other major life-limiting health conditions likely to cause death within 5 years.

The ASPREE study complies with the Declaration of Helsinki and was approved by multiple Institutional Review Boards (www.aspree.org). All participants signed written informed consent on participation. The present sub-study was approved by the Monash University Human Research Ethics Committee (MHREC 30734).

### Epigenetic age

Blood samples were processed within 4 h of the blood collection and stored at ultra-low − 80° temperatures. All samples have barcodes and are prepared for long-term storage at a purpose-built large biorepository near the ASPREE National Co-ordinating Centre in Melbourne (www.aspree.org).

DNA methylation (DNAm) from peripheral blood samples (buffy coat) collected at baseline was extracted using Qiagen DNeasy Blood and Tissue Kits (https://www.qiagen.com/au). Cytosine-phosphate-guanine (CpG) probe methylation was measured using the Illumina Infinium Methylation EPIC BeadChip (EPIC) and run at the Australian Genome Research Facility, Melbourne, Victoria (https://www.agrf.org.au/). The programming platform R version 4.1.3 with R package minfi was used for preprocessing data for use in Horvath’s online “New DNAm Age Calculator” (https://dnamage.genetics.ucla.edu/new). The preprocessNoob method was used to normalize the EPIC data [29]. According to the online age calculator’s expectation, the beta values from the EPIC data set were limited to the probes that the 450 K array could measure, and the beta values of missing probes that are not present on the 450 K array (33,124 probes) were filled with “NA” in the data set. The list of 33,124 missing probes has been described previously [30].

Next, the sample methylation data file (including beta measurements) and the sample annotation file, which included sample ID, age, sex, and tissue type, were uploaded to the online calculator. The default option (i.e., “Normalize Data”) and an additional option—“Advanced Analysis”—were selected before submitting the data. The output of the online age calculator consisted of HorvathAge [5], HannumAge [6], PhenoAge [7], GrimAge [8], and their AA measures (residuals from regressing epigenetic age on chronological age). A positive value of AA indicates an older epigenetic age relative to chronological age. Further, version-2 of GrimAge [11] and its AA were estimated through the new online software of Clock Foundation (https://dnamage.clockfoundation.org/). We also considered other DNAm estimates—telomere length [31] and DNAm-based components of original GrimAge and GrimAge2 [8, 11] (adrenomedullin (ADM), beta-2-microglobulin (B2M), cystatin-C, growth differentiation factor 15 (GDF-15), leptin, smoking-pack-years, plasminogen activation inhibitor 1 (PAI-1), tissue inhibitor of metalloproteinases 1 (TIMP-1), high sensitivity C-reactive protein (CRP), and hemoglobin A1C (A1C)). The Pace of Aging (DunedinPACE) is a measure of the ongoing rate of deterioration in system integrity. DunedinPACE was estimated using R software (version 4.1.3) and the codes described in Belsky et al. [9].

### Brain age

ASPREE participants living in the Greater Melbourne region and without MRI contraindications (e.g., specific foreign bodies or electronic implants, claustrophobia) were invited to participate in the ASPREE-NEURO sub-study [32]. Brain scans were performed at the Monash Biomedical Imaging Facility, Melbourne, Australia, using a 3 Tesla Siemens Skyra scanner (Siemens, Erlangen, Germany). Raw 3D T1-weighted images were pre-processed using the MATLAB Statistical Parametric Mapping software package (University College London, London, UK) [33]. Full details regarding the pre-processing of this data have been described in detail previously [19]. Brain age was calculated using a trained, publicly accessible processing pipeline and prediction algorithm created by Cole et al. (BrainageR, version 2.1, https://github.com/james-cole/brainageR) [16].

The brain-predicted age difference (brain-PAD) refers to the difference between an individual’s estimated brain age and their chronological age. A positive brain-PAD value indicates that the individual’s brain is older than its chronological age.

### Frailty index

A deficit accumulation frailty index (FI) of 67 items was constructed based on the method of Rockwood and Mitnitski [13]. The index included chronic disease diagnosis and indicators (24 items), physical deficits interfering in completing the activities of daily living (26 items), mental and psychosocial deficits (11 items), and measures of physical functional engagement and cognitive function (6 items), as described in detail previously [34]. Each item was scored from 0 (deficit absent) to 1 (deficit present). The FI was derived by summing the number of deficits noted for each person and dividing this by the total number of possible deficits. Higher scores indicate greater frailty.

### Sociodemographic characteristics

Sociodemographic characteristics included in this study were chronological age, sex (male or female), years of education (< 12 years or ≥ 12 years), living situation (at home alone or with family/others), and socioeconomic status (SES) (very low, low, middle, high, very high). SES was estimated through the Socio-Economic Indices for Areas-Index of Relative Socioeconomic Advantage and Disadvantage (SEIFA-IRSAD) based on the information from the 2011 Australian Census using the residential postcodes of participants [35]. Smoking was categorized into never, former, or current. Alcohol consumption was defined as never, former, current-low risk (i.e., ≤ 10 standard drinks per week and ≤ 4 standard drinks on any 1 day), or current-high risk (i.e., > 10 standard drinks per week or > 4 standard drinks on any 1 day) [36].

### Clinical measures, chronic conditions, and self-reported outcomes

Clinical measures included systolic blood pressure (SBP) and diastolic blood pressure (DBP) in mmHg, dominant hand grip strength in kilograms, and gait speed in meters/seconds. Full detailed procedures for taking measurements have been previously reported [37]. In brief, for SBP and DBP, the average scores after three measurements were used. The grip strength of both hands was assessed three times using an isometric handheld dynamometer (Jamar; Lafayette Instruments). The mean score for each participant’s self-reported dominant hand was used in the analysis. Gait speed was assessed as the time spent walking 3 m at a habitual pace on a flat surface from a standing start. Gait speed was measured twice, and the mean score was used in the analysis.

Self-reported outcomes were assessed through the SF-12 version-2 questionnaire [38]. Self-rated health ranged from 1 (excellent) to 5 (poor). Physical (PCS) and mental component scores (MCS) of health-related quality of life (HRQoL) were generated using a norm-based scoring method [38]. Higher scores indicate better health-related quality of life.

Chronic conditions investigated included hypertension (systolic blood pressure ≥ 140 mmHg or diastolic blood pressure ≥ 90 mmHg and/or treatment for high blood pressure), diabetes (fasting glucose ≥ 126 mg/dL or treatment or self-report), dyslipidemia (cholesterol-lowering medications or low-density lipoprotein, LDL > 160 mg/dL (> 4.1 mmol/L), or serum cholesterol ≥ 212 mg/dL (≥ 5.5 mmol/L)), obesity (body mass index ≥ 30 kg/m^2^) [39], chronic kidney disease (estimated glomerular filtration rate (eGFR) < 60 ml/min per 1.73 m^2^ or spot urinary albumin: creatinine ratio (UACR) ≥ 3 mg/mmol with eGFR ≥ 60 ml/min per 1.73 m^2^) [40], and depression (Center for Epidemiological Studies-Depression CES-D-10 score ≥ 8) [41].

### Statistical analysis

First, the correlations between different measures of biological age (i.e., HorvathAge, HannumAge, PhenoAge, GrimAge, GrimAge2, DunedinPACE, brain age, and FI) and chronological age were examined. Next, Pearson’s correlation, with pairwise deletion, was used to investigate the correlation between the different biological aging measures. Pearson’s correlation, *t*-tests, or one-way ANOVA, as appropriate, were also used to investigate the correlations between the AA measures and other DNAm-based estimates and sociodemographic characteristics, clinical measures, and chronic diseases, separately in males and females. Analyses were undertaken in STATA statistical software version 17.0, and the figures and graphs were plotted using the programming platform R version 4.1.3.

## Results

### Participants’ characteristics

The characteristics of the 560 study participants are shown in Table 1. Approximately, half were female (50.7%), and the chronological age ranged from 70.0 to 92.0 years, with a mean of 74.5 years. The estimated brain age (mean, 72.1 ± 6.9 years), HorvathAge (mean, 70.6 ± 6.1 years), GrimAge (mean, 70.1 ± 5.0 years), and GrimAge2 (mean, 74.1 ± 5.3 years) were similar to the mean chronological age, whereas HannumAge (mean, 59.5 ± 6.1 years) and PhenoAge (mean, 58.9 ± 7.7 years) were younger than the chronological age. DunedinPACE ranged from 0.69 to 1.30, with a mean of 0.97, and the mean FI was 0.10. Compared to females, males had significantly higher epigenetic aging whereas females had higher deficit accumulation frailty scores than males. Brain age was identical between males and females (Table 1).

**Table 1:** The characteristics of study participants

	All participants (*n* = 560)	Males (*n* = 276, 49.3%)	Females (*n* = 284, 50.7%)
Chronological age (years); mean (SD)	74.5 (4.2)	74.5 (4.2)	74.5 (4.2)
Epigenetic age (years)			
HorvathAge; mean (SD)	70.6 (6.1)	71.0 (6.1)	70.1 (6.0)
HannumAge; mean (SD)	59.5 (6.1)	60.4 (6.1)	58.6 (6.0)
PhenoAge; mean (SD)	58.9 (7.7)	59.7 (7.7)	58.1 (7.6)
GrimAge; mean (SD)	70.1 (5.0)	71.5 (5.1)	68.6 (4.5)
GrimAge2; mean (SD)	74.1 (5.3)	75.4 (5.5)	72.8 (4.8)
DunedinPACE; mean (SD)	0.97 (0.11)	0.99 (0.11)	0.95 (0.11)
Epigenetic age acceleration (AA, years)			
HorvathAA; mean (SD)	0.08 (4.96)	0.49 (4.96)	− 0.31 (4.92)
HannumAA; mean (SD)	0.07 (4.84)	0.79 (5.04)	− 0.64 (4.54)
PhenoAA; mean (SD)	0.09 (6.37)	0.76 (6.46)	− 0.55 (6.21)
GrimAA; mean (SD)	0.02 (3.52)	1.35 (3.34)	− 1.27 (3.19)
Grim2AA; mean (SD)	0.02 (4.04)	1.16 (3.99)	− 1.08 (3.78)
Other measures of biological aging			
Mean brain age^a^; mean (SD)	72.1 (6.9)	72.1 (7.4)	72.1 (6.4)
Brain-PAD^a^; mean (SD)	− 1.50 (6.05)	− 1.70 (6.39)	− 1.27 (5.67)
Deficit accumulation frailty index; mean (SD)	0.10 (0.06)	0.09 (0.06)	0.12 (0.06)
Participants’ characteristics			
Years of education; *n* (%)			
< 12 years	234 (41.8)	111 (40.2)	123 (43.3)
≥ 12 years	326 (58.2)	165 (59.8)	161 (56.7)
Living situation; *n* (%)			
At home alone	159 (28.4)	47 (17.0)	112 (39.4)
With family or others	401 (71.6)	229 (83.0)	172 (60.6)
Socioeconomic status (SES)^b^; *n* (%)			
Very low	48 (8.6)	23 (8.4)	25 (8.8)
Low	44 (7.9)	19 (6.9)	25 (8.8)
Middle	85 (15.2)	43 (15.6)	42 (14.8)
High	121 (21.7)	61 (22.2)	60 (21.1)
Very high	261 (46.7)	129 (46.9)	132 (46.5)
Smoking; *n* (%)			
Never	312 (55.7)	129 (46.7)	183 (64.4)
Former	227 (40.5)	135 (48.9)	92 (32.4)
Current	21 (3.8)	12 (4.4)	9 (3.2)
Alcohol consumption, *n* (%)			
Never	80 (14.3)	25 (9.1)	55 (19.4)
Former	21 (3.8)	12 (4.4)	9 (3.2)
Current-low risk^c^	336 (60.0)	158 (57.3)	178 (62.7)
Current-high risk^d^	123 (22.0)	81 (29.4)	42 (14.8)
Clinical measures			
Systolic blood pressure (mmHg); mean (SD)	137.8 (16.0)	140.7 (15.3)	134.9 (16.2)
Diastolic blood pressure (mmHg); mean (SD)	75.8 (10.2)	77.3 (10.1)	74.2 (10.0)
Grip strength (kg)^e^; mean (SD)	28.5 (9.9)	35.9 (7.6)	21.3 (5.6)
Gait speed (m/s); mean (SD)	1.1 (0.2)	1.1 (0.2)	1.0 (0.2)
Self-reported health outcomes			
Self-rated health; *n* (%)			
Excellent	89 (15.9)	43 (15.6)	46 (16.2)
Very good	279 (49.8)	133 (48.2)	146 (51.4)
Good	172 (30.7)	90 (32.6)	82 (28.9)
Fair	19 (3.4)	10 (3.6)	9 (3.2)
Poor	1 (0.2)	0 (0.0)	1 (0.4)
Health-related quality of life			
Physical component score; mean (SD)	48.5 (8.7)	49.3 (8.1)	47.7 (9.1)
Mental component score; mean (SD)	55.5 (7.4)	56.1 (6.4)	54.9 (8.2)
Chronic conditions			
Hypertension; *n* (%)			
Yes	381 (68.0)	197 (71.4)	184 (64.8)
No	179 (32.0)	79 (28.6)	100 (35.2)
Diabetes; *n* (%)			
Yes	65 (11.6)	43 (15.6)	22 (7.8)
No	495 (88.4)	233 (84.4)	262 (92.3)
Dyslipidemia; *n* (%)			
Yes	351 (62.7)	140 (50.7)	211 (74.3)
No	209 (37.3)	136 (49.3)	73 (25.7)
Obesity^f^; *n* (%)			
Yes	151 (27.1)	70 (25.6)	81 (28.6)
No	406 (72.9)	204 (74.5)	202 (71.4)
Chronic kidney disease^g^; *n* (%)			
Yes	107 (20.4)	53 (20.4)	54 (20.4)
No	418 (79.6)	207 (79.6)	211 (79.6)
Depression; *n* (%)			
Yes	57 (10.2)	26 (9.4)	31 (10.9)
No	503 (89.8)	250 (90.6)	253 (89.1)

^a^Numbers of participants, total *n* = 400, males *n* = 209, and females *n* = 191

^b^SES, total *n* = 559, males *n* = 275, and females *n* = 284

^c^Current-low risk, ≤ 10 standard drinks per week and ≤ 4 standard drinks on any 1 day

^d^Current-high risk, > 10 standard drinks per week or > 4 standard drinks on any 1 day

^e^Grip strength, total *n* = 558, males *n* = 275, and females *n* = 283

^f^Obesity, total *n* = 557, males *n* = 274, and females *n* = 283

^g^Chronic kidney disease, total *n* = 525, males *n* = 260, and females *n* = 265

### Correlation between different measures of biological age, and chronological age

**Fig. 1:**
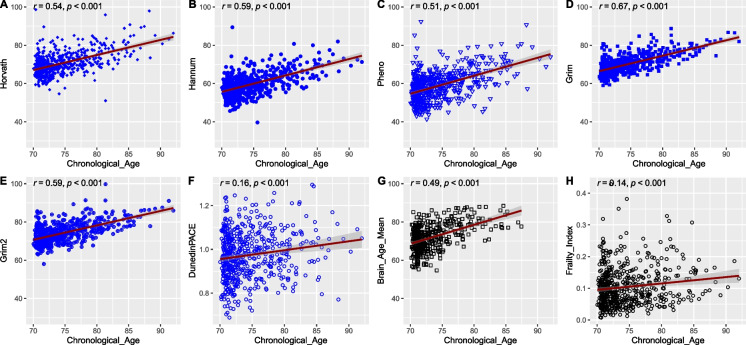
Correlations between estimated biological age (y-axis) and chronological age (x-axis). **A** HorvathAge vs. chronological age (*n* = 560); **B** HannumAge vs. chronological age (*n* = 560); **C** PhenoAge vs. chronological age (*n* = 560); **D** GrimAge vs. chronological age (*n* = 560); **E** GrimAge2 vs. chronological age (*n* = 560); **F** DunedinPACE vs. chronological age (*n* = 560); **G** Brain age vs. chronological age (*n* = 400); **H** deficit accumulation frailty index vs. chronological age (*n* = 560)

Epigenetic age, brain age, and FI were positively correlated with chronological age (Fig. 1). The strongest correlation was found for GrimAge (*r* = 0.67), while DunedinPACE (*r* = 0.16) and FI (*r* = 0.14) had the weakest correlations with chronological age.

### Correlation between AA, brain-PAD, and FI

**Fig. 2:**
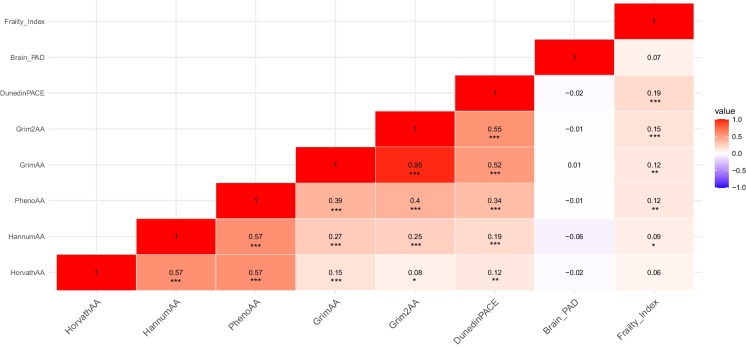
Pearson’s correlation matrix on epigenetic age acceleration (HorvathAA, HannumAA, PhenoAA, GrimAA, Grim2AA, DunedinPACE), brain-PAD, and deficit accumulation frailty index. *p-value < 0.05; **p-value < 0.01; ***p-value < 0.001

All AA measures were positively correlated with one another (*r* ranged from 0.08 to 0.57), but DunedinPACE showed weak correlations, particularly with 1st generation HorvathAA and HannumAA (Fig. 2). GrimAA and Grim2AA were strongly correlated with each other (*r* = 0.95). Brain-PAD was not correlated with any of the AA measures, or FI. FI had the strongest correlation with DunedinPACE (*r* = 0.19) and Grim2AA (*r* = 0.15), and significant but weak correlations with HannumAA, PhenoAA, and GrimAA (*r* ≤ 0.12). Similar correlations were also found in males and females (Supplementary Tables 1 and 2).

### AA variations according to the sociodemographic characteristics

In males (Supplementary Table 3), we observed an association between smoking and both versions of GrimAA (*p*-value < 0.001) and DunedinPACE only (*p*-value = 0.003). In females, however (Supplementary Table 4), we observed associations across all the sociodemographic characteristics. In particular, less than 12 years of education was associated with accelerated DunedinPACE; living alone was linked to an accelerated HorvathAA, low SES, current smoking, and former alcohol consumption were associated with higher in both versions of GrimAA (*p*-values < 0.05).

### Correlation between AA, clinical measures, and self-reported health outcomes

**Fig. 3:**
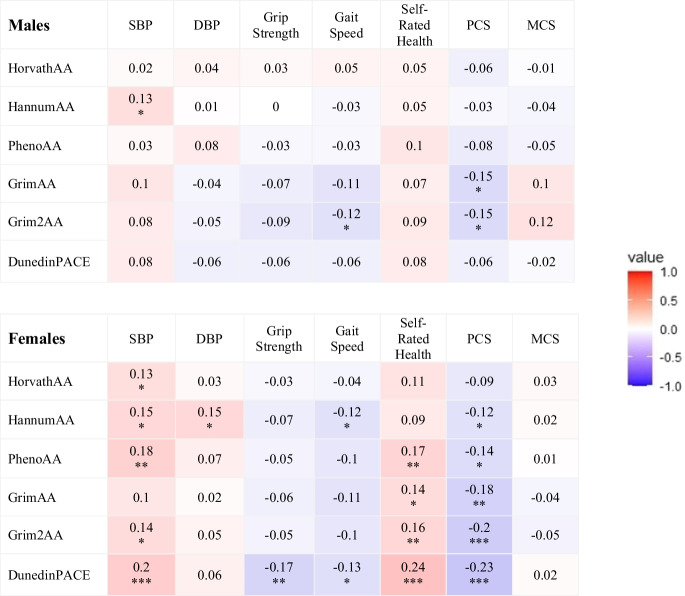
Correlation matrix between epigenetic age acceleration measures and systolic blood pressure (SBP), diastolic blood pressure (DBP), grip strength, gait speed, self-rated health (higher scores indicating worse health), and physical component score (PCS) and mental component score (MCS) of the SF-12, separately in males and females. *p-value < 0.05; **p-value < 0.01; ***p-value < 0.001

Correlations between AA measures and clinical measures/self-reported health outcomes were also more commonly found in females than males (Fig. 3). In males, correlations were observed between SBP and HannumAA (*r* = 0.13), between gait speed and Grim2AA (*r* =  − 0.12), and between PCS and both versions of GrimAA (*r* =  − 0.15). In females, all clinical measures and self-reported health status, except for MCS, were correlated with AA measures (*r* ranged from − 0.23 to 0.24) with DunedinPACE having the greatest number of significant correlations.

### AA variations according to the chronic condition status

Hypertension, diabetes, and chronic kidney disease were associated with accelerated GrimAA (both versions) and DunedinPACE in males (Supplementary Table 5). In contrast, only obesity and depression were associated with accelerated GrimAA (both versions) in females, and obesity was also associated with accelerated DunedinPACE.

### Other DNAm-based estimates and their associations with sociodemographic characteristics, clinical measures, self-reported health outcomes, and the chronic condition status

There were also sex differences in the associations between other DNA-based estimates (telomere length, plasma proteins, and smoking-pack-years) and sociodemographic factors and health markers (Supplementary Tables 6 to 15). The correlations between telomere length and clinical measures except for DBP and MCS were found only in females (Supplementary Table 8). Regarding the chronic conditions, only chronic kidney disease was associated with shorter telomere length in females, and no associations were found in males (Supplementary Table 9). Correlations between either plasma proteins or smoking-pack-years and clinical measures were commonly found in both males and females (Supplementary Tables 12 and 13). At least one plasma protein was associated with diabetes, dyslipidemia, obesity, and chronic kidney disease in both males and females, but associations were more commonly found in males than females (Supplementary Tables 14 and 15).

## Discussion

In this study, we examined the extent to which different measures of epigenetic aging were correlated with a system-wide measure of biological aging (FI) and an organ-specific biological aging marker (brain-PAD), separately in males and females. We observed that FI had weak but significant positive correlations with AA in both sexes, whereas brain-PAD was not correlated with any of the other biological aging measures. This suggests that these system-wide measures of biological aging are capturing somewhat overlapping, yet distinct aspects of the aging process. We also found that epigenetic aging was correlated with participants’ sociodemographic characteristics and health status, but this varied between males and females. Together, these findings indicate that markers of biological aging may be useful clinical indicators of overall physical, cognitive, and psychosocial deficits even in relatively healthy older people who have reached 70 years without major illness, including CVD and dementia. Further, we also found that there were differences between males and females in the relationships between other DNAm estimates (i.e., telomere length, plasma proteins, and smoking-pack-years) and health status.

Not surprisingly, in our present study, the first-generation AA measures were most strongly correlated with one another, and the most recent of these measures, DunedinPACE, was most significantly associated with the second-generation AA measures where the correlation with Grim2AA was the highest. Our finding is supported by previous research [5, 6, 8, 9] and aligns with the general purpose for which these epigenetic clock algorithms were developed. The first-generation epigenetic clocks were generated to predict chronological age [5, 6] whereas the second-generation clocks, like GrimAge [8, 11], go beyond just estimating chronological age and additionally aim to predict the risk of developing health outcomes. To reflect that purpose, GrimAge algorithms were constructed by incorporating additional age-related clinical and laboratory measures as well as lifestyle factors such as smoking [8, 11]. Thus, clear evidence has been found that second-generation clocks outperform first-generation clocks in the risk prediction of age-related diseases and death [8]. The latest generation clock—DunedinPACE—was specifically designed to capture the gradual, progressive deterioration simultaneously affecting different organ systems with aging [9]. This noticeably contrasts with first-generation clocks which were designed and trained to predict chronological age (HorvathAge [5] and HannumAge [6]). Moreover, prior studies have reported significant but weak correlations between AA and system-wide FI [21, 42]. However, as ours is the first to investigate correlations with GrimAge2 and DunedinPACE, we added new evidence to the field that DunedinPACE and GrimAge2 are more strongly correlated with FI rather than other AAs. This is not surprising given both DunedinPACE and FI have a multidimensional nature. Like DunedinPACE, the FI defines biological aging through the accumulation of age-related health deficits across a range of functions (physical, psychological, and social). Noticeably, our study also demonstrated that the correlations of GrimAge2 with DunedinPACE and FI were slightly stronger than those of the original GrimAge. This finding could be due to the additional inclusion of two commonly used biomarkers (i.e., DNAm-based high-sensitivity CRP, and hemoglobin A1C) in the GrimAge2 development [11]. Further, our finding of GrimAge2 also somewhat reflects the fact that GrimAge2 outperforms the original GrimAge in terms of mortality and morbidity risk prediction [11].

Our finding that males had accelerated epigenetic age in comparison to females is consistent with prior evidence demonstrating that, for individuals with the same chronological age, males tend to be biologically older than females [43]. This also aligns with the established longer life expectancy of females compared to males [23] which could be driven by a complex interaction of biological factors, including genetic influences and sex hormones, as well as behavioral factors [44]. Males may be more likely to engage in risky behaviors and unhealthy lifestyles such as smoking and heavy drinking [45, 46] and have an age-related decline in testosterone concentrations, which could lead to a greater risk of fatal illnesses including heart disease and cancer [47, 48]. There is also evidence that females are more willing to engage with health-related information and are more likely to seek medical treatment for their illness [49] which together could help initiate early detection and intervention. In this manner, females may have less fatal outcomes. In contrast, females in our study had higher deficit accumulation FI scores than males, which captures the overall disease burden. This finding also supports the conclusion of a prior meta-analysis among 37,426 participants, showing that females of all ages have a poorer health status in terms of FI, compared to males [50]. Thus, our finding from this relatively healthy older cohort might also reflect the male–female health-survival paradox, in that females live longer and are more likely to experience disabilities and disease burdens [46, 51].

Several studies have investigated whether AA measures are associated with participants’ sociodemographic characteristics and markers of their health status [52–54]. These studies have generally, but not consistently, shown that lower SES status and/or risk factors for chronic diseases, such as high blood pressure and smoking, are associated with accelerated aging. However, very few studies have considered whether these associations may vary between males and females. Our study provides an important new contribution to this field, demonstrating clear evidence of sex differences. In females, several significant correlations were observed between AA and health risk factors such as low SES and living alone, as well as markers of physical functioning (e.g., grip strength and gait speed), SBP, DBP, and self-rated overall and physical health whereas only associations between AA, especially Grim2AA, and SBP, gait speed, and self-rated physical health were found in males. Thus, our cross-sectional findings highlight that accelerated epigenetic aging could be linked with physical health deterioration in both males and females. Hence, a further longitudinal study is suggested to enhance this novel relationship and to examine whether accelerated epigenetic aging could be predictive of physical health deterioration in later life. Moreover, significant associations were found between newer accelerated AA measures and hypertension, diabetes, and chronic kidney disease in males only whereas the associations of AA measures with obesity and depression were observed only in females. Although the exact reasons for these differences are still unclear, biological disparities and previously noted sex differences in the prevalence of health conditions [55, 56] may have contributed to our results. Overall, it highlights the importance of considering separately males and females when investigating causal inference of our observed relationships in future longitudinal studies.

Our study had both strengths and limitations. The strength of this study includes investigating several epigenetic clocks including the most recent ones—GrimAge2 and DunedinPACE and their correlations with the system-wide FI, and brain-PAD, among community-based individuals. Our sex-balanced sample (females 50.7%) allowed us to investigate the relationships between AA and markers of health in males and females separately. We acknowledged that our community-based sample comprises relatively healthy older Australians without dementia or CVD, and about two-thirds had high SES. Thus, this could limit the generalizability of our findings. However, the sample had a good distribution in terms of education level (e.g., 41.8% had < 12 years of education) and ranged in age from 70 to 92 years. The findings add new evidence about the utility of biological aging measures even in relatively healthy older individuals.

In conclusion, our study showed that, in relatively healthy older individuals, epigenetic age was correlated with the system-wide deficit accumulation FI, but not with the neuroimaging-based brain aging biomarker (brain-PAD). We also showed that despite having similar chronological ages, females in our study had lower epigenetic aging, but higher FI, than males. Further, this study provides novel evidence showing sex differences in the relationships between AA measures and markers of health status. Thus, our results highlight the importance of considering sex differences when investigating biological aging and its utility as a marker of health or disease risk.

### Supplementary Information

Below is the link to the electronic supplementary material.

Supplementary file1 (DOCX 50 KB)

Supplementary file2 (DOCX 86 KB)

## Data Availability

The data that support the findings of this study are available from authors upon reasonable request and with permission of the ASPREE principal investigators through the website (www.ASPREE.org). Data will be shared to approved researchers through a secure web-based data portal Safe Haven.
