# Supplementary material for: Sex differences in biological aging and the association with clinical measures in older adults
Source: GeroScience. 2023 Sep 25;46(2):1775–88. doi: 10.1007/s11357-023-00941-z (PMC10828143; doi:10.1007/s11357-023-00941-z)
Supplement: Supplementary file 1 — Supplementary file1 (DOCX 50 KB) [file 11357_2023_941_MOESM1_ESM.docx]

**Supplementary Table 1** Pearson's correlation matrix for epigenetic age acceleration measures (HorvathAA, HannumAA, PhenoAA, GrimAA, Grim2AA, and DunedinPACE), brain-PAD, and deficit accumulation frailty index in males

|  | **HorvathAA** | **HannumAA** | **PhenoAA** | **GrimAA** | **Grim2AA** | **DunedinPACE** | **Brain-PAD** | **Frailty Index** |
| --- | --- | --- | --- | --- | --- | --- | --- | --- |
| **HorvathAA** |  |  |  |  |  |  |  |  |
| **HannumAA** | 0.57 **<0.001** |  |  |  |  |  |  |  |
| **PhenoAA** | 0.57 **<0.001** | 0.54 **<0.001** |  |  |  |  |  |  |
| **GrimAA** | 0.13 **0.03** | 0.19 **0.002** | 0.32 **<0.001** |  |  |  |  |  |
| **Grim2AA** | 0.02  0.74 | 0.15  **0.01** | 0.29  **<0.001** | 0.94  **<0.001** |  |  |  |  |
| **DunedinPACE** | 0.11 0.08 | 0.11 0.07 | 0.27 **<0.001** | 0.50 **<0.001** | 0.54  **<0.001** |  |  |  |
| **Brain-PAD** | -0.02 0.80 | -0.09 0.19 | -0.02 0.79 | -0.05 0.47 | -0.08  0.23 | -0.06 0.41 |  |  |
| **Frailty Index** | 0.10 0.10 | 0.11 0.06 | 0.15 **0.01** | 0.20 **0.001** | 0.19  **0.001** | 0.21 **<0.001** | 0.06 0.43 |  |

Bold text indicates significant correlations.

**Supplementary Table 2** Pearson's correlation matrix for epigenetic age acceleration measures (HorvathAA, HannumAA, PhenoAA, GrimAA, Grim2AA, and DunedinPACE), brain-PAD, and deficit accumulation frailty index in females

|  | **HorvathAA** | **HannumAA** | **PhenoAA** | **GrimAA** | **Grim2AA** | **DunedinPACE** | **Brain-PAD** | **Frailty Index** |
| --- | --- | --- | --- | --- | --- | --- | --- | --- |
| **HorvathAA** |  |  |  |  |  |  |  |  |
| **HannumAA** | 0.57 **<0.001** |  |  |  |  |  |  |  |
| **PhenoAA** | 0.56 **<0.001** | 0.60 **<0.001** |  |  |  |  |  |  |
| **GrimAA** | 0.12 0.05 | 0.29 **<0.001** | 0.44 **<0.001** |  |  |  |  |  |
| **Grim2AA** | 0.11  0.07 | 0.30  **<0.001** | 0.50  **<0.001** | 0.95  **<0.001** |  |  |  |  |
| **DunedinPACE** | 0.10 0.08 | 0.23 **<0.001** | 0.39 **<0.001** | 0.50 **<0.001** | 0.53  **<0.001** |  |  |  |
| **Brain-PAD** | -0.02 0.77 | -0.01 0.85 | 0.01 0.90 | 0.12 0.11 | 0.12  0.09 | 0.05 0.50 |  |  |
| **Frailty Index** | 0.06 0.28 | 0.13 **0.03** | 0.14 **0.02** | 0.22 **<0.001** | 0.24  **<0.001** | 0.24 **<0.001** | 0.09 0.22 |  |

Bold text indicates significant correlations.

**Supplementary Table 3** Epigenetic age acceleration measures (HorvathAA, HannumAA, PhenoAA, GrimAA, Grim2AA, and DunedinPACE) according to characteristics of males in the study (n = 276)

|  | **HorvathAA**  **Mean (SD)** | **HannumAA**  **Mean (SD)** | **PhenoAA**  **Mean (SD)** | **GrimAA**  **Mean (SD)** | **Grim2AA**  **Mean (SD)** | **DunedinPACE**  **Mean (SD)** |
| --- | --- | --- | --- | --- | --- | --- |
| **Years of education** |  |  |  |  |  |  |
| <12 years | 0.84 (4.94) | 0.64 (4.92) | 0.66 (5.81) | 1.51 (3.16) | 1.29 (3.61) | 1.00 (0.11) |
| ≥12 years | 0.26 (4.98) | 0.88 (5.13) | 0.83 (6.88) | 1.24 (3.46) | 1.08 (4.24) | 0.99 (0.10) |
| **P-value** | 0.34 | 0.70 | 0.83 | 0.50 | 0.67 | 0.51 |
| **Living situation** |  |  |  |  |  |  |
| At home alone | 1.49 (3.66) | 0.15 (5.16) | 1.57 (5.13) | 1.77 (3.67) | 1.38 (4.50) | 1.00 (0.09) |
| With family or others | 0.29 (5.17) | 0.92 (5.01) | 0.60 (6.70) | 1.26 (3.27) | 1.12 (3.89) | 0.99 (0.11) |
| **P-value** | 0.13 | 0.34 | 0.35 | 0.34 | 0.68 | 0.60 |
| **Socioeconomic status (SES)** |  |  |  |  |  |  |
| Very low | 2.19 (5.31) | 2.07 (4.71) | 2.31 (4.53) | 2.70 (2.61) | 2.62 (3.34) | 1.02 (0.12) |
| Low | 1.79 (3.99) | 0.27 (3.98) | -0.53 (5.40) | 0.99 (2.43) | 0.33 (3.03) | 1.00 (0.09) |
| Middle | -0.31 (4.99) | 0.16 (5.88) | -0.70 (6.49) | 1.08 (3.44) | 0.97 (4.19) | 0.98 (0.10) |
| High | 0.74 (4.85) | 0.75 (4.91) | 1.16 (6.09) | 1.92 (3.60) | 1.94 (4.41) | 0.99 (0.10) |
| Very high | 0.16 (5.05) | 0.84 (5.03) | 0.95 (7.02) | 0.95 (3.34) | 0.70 (3.89) | 0.99 (0.11) |
| **P-value** | 0.21 | 0.67 | 0.33 | 0.09 | 0.09 | 0.68 |
| **Smoking** |  |  |  |  |  |  |
| Never | 0.78 (4.69) | 1.08 (4.35) | 0.76 (6.62) | 0.24 (2.48) | 0.02 (3.07) | 0.97 (0.10) |
| Former | 0.29 (5.36) | 0.50 (5.68) | 0.71 (6.46) | 1.81 (3.20) | 1.66 (4.07) | 1.00 (0.10) |
| Current | -0.22 (2.85) | 0.86 (4.27) | 1.40 (5.08) | 8.09 (3.91) | 7.84 (4.59) | 1.07 (0.12) |
| **P-value** | 0.64 | 0.65 | 0.94 | **<0.001** | **<0.001** | **0.003** |
| **Alcohol consumption** |  |  |  |  |  |  |
| Never | 0.93 (4.84) | 1.17 (4.26) | 2.13 (7.49) | 1.64 (3.32) | 1.67 (3.93) | 1.01 (0.10) |
| Former | 3.53 (5.17) | 3.88 (5.43) | 2.87 (5.66) | 1.67 (3.07) | 1.34 (3.20) | 1.03 (0.07) |
| Current-Low Risk | 0.33 (4.99) | 0.58 (5.28) | 0.34 (6.59) | 1.00 (3.29) | 0.83 (3.97) | 0.98 (0.10) |
| Current-High Risk | 0.24 (4.86) | 0.61 (4.62) | 0.86 (5.95) | 1.89 (3.44) | 1.64 (4.17) | 1.00 (0.11) |
| **P-value** | 0.17 | 0.17 | 0.38 | 0.24 | 0.45 | 0.41 |

P-values were based on t-tests or one-way ANOVA tests, as appropriate. Bold text indicates significant associations.

**Supplementary Table 4** Epigenetic age acceleration measures (HorvathAA, HannumAA, PhenoAA, GrimAA, Grim2AA, and DunedinPACE) according to characteristics of females in the study (n = 284)

|  | **HorvathAA**  **Mean (SD)** | **HannumAA**  **Mean (SD)** | **PhenoAA**  **Mean (SD)** | **GrimAA**  **Mean (SD)** | **Grim2AA**  **Mean (SD)** | **DunedinPACE**  **Mean (SD)** |
| --- | --- | --- | --- | --- | --- | --- |
| **Years of education** |  |  |  |  |  |  |
| <12 years | -0.68 (4.37) | -0.81 (4.83) | -0.25 (6.07) | -1.03 (3.31) | -0.72 (4.00) | 0.97 (0.11) |
| ≥12 years | -0.03 (5.30) | -0.50 (4.32) | -0.79 (6.33) | -1.46 (3.10) | -1.36 (3.59) | 0.94 (0.10) |
| **P-value** | 0.27 | 0.57 | 0.47 | 0.26 | 0.16 | **0.03** |
| **Living situation** |  |  |  |  |  |  |
| At home alone | 0.51 (5.33) | -0.37 (4.82) | -0.16 (7.00) | -1.32 (3.24) | -1.23 (3.79) | 0.96 (0.12) |
| With family or others | -0.85 (4.58) | -0.81 (4.36) | -0.81 (5.65) | -1.25 (3.17) | -0.99 (3.78) | 0.95 (0.10) |
| **P-value** | **0.02** | 0.42 | 0.39 | 0.86 | 0.61 | 0.56 |
| **Socioeconomic status (SES)** |  |  |  |  |  |  |
| Very low | -1.13 (3.94) | 0.00 (4.71) | 1.97 (5.88) | -0.32 (2.82) | 0.82 (3.72) | 1.02 (0.11) |
| Low | -0.71 (3.71) | -1.75 (3.88) | -0.52 (5.24) | -0.71 (4.65) | -0.58 (5.42) | 0.97 (0.13) |
| Middle | -0.65 (4.00) | -1.03 (4.78) | -0.56 (5.84) | -0.71 (3.39) | -0.61 (3.83) | 0.94 (0.11) |
| High | 0.06 (4.48) | 0.02 (4.83) | -0.76 (5.95) | -0.92 (3.41) | -0.90 (4.01) | 0.95 (0.10) |
| Very high | -0.15 (5.72) | -0.72 (4.42) | -0.94 (6.64) | -1.91 (2.64) | -1.78 (3.13) | 0.95 (0.11) |
| **P-value** | 0.82 | 0.46 | 0.32 | **0.03** | **0.02** | 0.05 |
| **Smoking** |  |  |  |  |  |  |
| Never | -0.06 (5.11) | -0.69 (4.53) | -0.88 (6.40) | -2.09 (2.54) | -1.98 (3.18) | 0.95 (0.11) |
| Former | -0.66 (4.61) | -0.56 (4.63) | 0.26 (5.94) | -0.31 (3.24) | 0.02 (3.82) | 0.96 (0.10) |
| Current | -1.90 (3.90) | -0.33 (4.37) | -2.24 (4.26) | 5.56 (4.03) | 5.89 (4.74) | 1.03 (0.08) |
| **P-value** | 0.40 | 0.96 | 0.25 | **<0.001** | **<0.001** | 0.09 |
| **Alcohol consumption** |  |  |  |  |  |  |
| Never | 0.10 (4.44) | 0.43 (4.61) | -0.53 (6.77) | -1.25 (3.36) | -1.15 (4.09) | 0.95 (0.11) |
| Former | -0.33 (5.71) | -1.96 (3.70) | -0.21 (5.47) | 2.45 (5.18) | 2.56 (5.93) | 1.02 (0.11) |
| Current-Low Risk | -0.13 (5.16) | -0.68 (4.71) | -0.40 (6.27) | -1.39 (3.06) | -1.20 (3.59) | 0.96 (0.11) |
| Current-High Risk | -1.63 (4.22) | -1.54 (3.66) | -1.31 (5.48) | -1.60 (2.60) | -1.26 (3.32) | 0.94 (0.10) |
| **P-value** | 0.30 | 0.14 | 0.86 | **0.004** | **0.03** | 0.22 |

P-values were based on t-tests or one-way ANOVA tests, as appropriate. Bold text indicates significant associations.

**Supplementary Table 5** Epigenetic aging according to chronic conditions

|  | **Males n = 276** | | | | | |  | **Females n = 284** | | | | | |
| --- | --- | --- | --- | --- | --- | --- | --- | --- | --- | --- | --- | --- | --- |
|  | **HorvathAA** | **HannumAA** | **PhenoAA** | **GrimAA** | **Grim2AA** | **DunedinPACE** |  | **HorvathAA** | **HannumAA** | **PhenoAA** | **GrimAA** | **Grim2AA** | **DunedinPACE** |
|  | **Mean**  **(SD)** | **Mean**  **(SD)** | **Mean**  **(SD)** | **Mean (SD)** | **Mean (SD)** | **Mean**  **(SD)** |  | **Mean**  **(SD)** | **Mean**  **(SD)** | **Mean (SD)** | **Mean (SD)** | **Mean (SD)** | **Mean**  **(SD)** |
| **Hypertension** | |  |  |  |  |  |  |  |  |  |  |  |  |
| Yes | 0.56  (4.76) | 1.05  (4.64) | 1.10  (6.20) | 1.61 (3.34) | 1.49  (3.76) | 1.00  (0.10) |  | 0.09  (5.33) | -0.37  (4.67) | -0.22  (6.20) | -1.11  (3.11) | -0.85  (3.69) | 0.96  (0.11) |
| No | 0.34  (5.47) | 0.12  (5.90) | -0.07  (7.05) | 0.68 (3.27) | 0.35  (4.44) | 0.97  (0.11) |  | -1.05  (3.99) | -1.13  (4.28) | -1.18  (6.23) | -1.58  (3.33) | -1.51  (3.93) | 0.94  (0.11) |
| **P-value** | 0.74 | 0.16 | 0.17 | **0.04** | **0.03** | **0.04** |  | 0.06 | 0.17 | 0.22 | 0.23 | 0.17 | 0.06 |
| **Diabetes** |  |  |  |  |  |  |  |  |  |  |  |  |  |
| Yes | 2.22  (5.12) | 2.17  (4.00) | 3.00  (6.75) | 2.77 (3.73) | 3.10  (3.83) | 1.04  (0.08) |  | -0.61  (3.85) | -0.20  (4.15) | -0.50  (5.79) | -0.94  (3.60) | -0.37  (4.52) | 0.98  (0.07) |
| No | 0.18  (4.88) | 0.53  (5.17) | 0.35  (6.34) | 1.08 (3.20) | 0.81  (3.93) | 0.98  (0.11) |  | -0.29  (5.01) | -0.67  (4.58) | -0.56  (6.26) | -1.30  (3.16) | -1.14  (3.71) | 0.95  (0.11) |
| **P-value** | **0.01** | 0.05 | **0.01** | **0.002** | **<0.001** | **<0.001** |  | 0.77 | 0.64 | 0.97 | 0.61 | 0.35 | 0.25 |
| **Dyslipidemia** |  |  |  |  |  |  |  |  |  |  |  |  |  |
| Yes | 0.49  (4.76) | 0.56  (4.25) | 1.08  (6.37) | 1.54 (3.38) | 1.40  (3.97) | 1.00  (0.10) |  | -0.19  (5.20) | -0.61  (4.81) | -0.78  (6.30) | -1.44  (3.19) | -1.33  (3.76) | 0.95  (0.11) |
| No | 0.50  (5.19) | 1.02  (5.74) | 0.44  (6.56) | 1.15 (3.29) | 0.92  (4.02) | 0.98  (0.11) |  | -0.67  (4.05) | -0.72  (3.69) | 0.10  (5.96) | -0.79  (3.16) | -0.39  (3.76) | 0.97  (0.10) |
| **P-value** | 0.98 | 0.45 | 0.41 | 0.33 | 0.32 | 0.09 |  | 0.47 | 0.85 | 0.30 | 0.13 | 0.07 | 0.15 |
| **Obesity ^a^** |  |  |  |  |  |  |  |  |  |  |  |  |  |
| Yes | 0.64  (4.57) | 0.51  (5.47) | 0.34  (6.00) | 1.65 (3.24) | 1.69  (3.56) | 1.01  (0.10) |  | -0.50  (5.09) | -0.42  (4.35) | 0.64  (5.69) | -0.59  (2.97) | -0.12  (3.78) | 0.99  (0.10) |
| No | 0.45  (5.11) | 0.88  (4.91) | 0.93  (6.65) | 1.24 (3.38) | 0.98  (4.14) | 0.99  (0.10) |  | -0.22  (4.87) | -0.70  (4.62) | -0.97  (6.31) | -1.52  (3.23) | -1.43  (3.69) | 0.94  (0.11) |
| **P-value** | 0.79 | 0.59 | 0.52 | 0.38 | 0.20 | 0.05 |  | 0.66 | 0.64 | 0.05 | **0.03** | **0.01** | **0.002** |
| **Chronic Kidney Disease ^b^** | |  |  |  |  |  |  |  |  |  |  |  |  |
| Yes | -0.15  (5.57) | 1.47  (3.92) | 1.44  (5.88) | 2.99 (3.56) | 3.54  (4.30) | 1.04  (0.10) |  | -0.25  (5.87) | -0.23  (4.95) | -0.13  (5.92) | -1.19  (3.07) | -0.87  (3.56) | 0.97  (0.11) |
| No | 0.64  (4.77) | 0.55  (5.28) | 0.62  (6.62) | 0.90 (3.13) | 0.53  (3.67) | 0.98  (0.10) |  | -0.35  (4.74) | -0.62  (4.51) | -0.79  (6.30) | -1.40  (3.10) | -1.28  (3.71) | 0.95  (0.11) |
| **P-value** | 0.30 | 0.24 | 0.41 | **<0.001** | **<0.001** | **<0.001** |  | 0.90 | 0.58 | 0.49 | 0.64 | 0.47 | 0.35 |
| **Depression** |  |  |  |  |  |  |  |  |  |  |  |  |  |
| Yes | 0.02  (3.97) | 1.43  (3.22) | 1.31  (4.99) | 1.69 (2.78) | 1.57  (3.45) | 1.01  (0.11) |  | -0.45  (4.72) | -1.05  (4.60) | 0.10  (6.23) | 0.30  (4.73) | 0.61  (5.18) | 0.96  (0.10) |
| No | 0.54  (5.06) | 0.72  (5.19) | 0.71  (6.60) | 1.31 (3.39) | 1.12  (4.05) | 0.99  (0.10) |  | -0.30  (4.96) | -0.59  (4.54) | -0.63  (6.22) | -1.47  (2.91) | -1.29  (3.53) | 0.95  (0.11) |
| **P-value** | 0.61 | 0.49 | 0.65 | 0.58 | 0.58 | 0.41 |  | 0.87 | 0.60 | 0.54 | **0.003** | **0.01** | 0.73 |

^a^ Obesity, Males N = 274, Females N = 283;

^b^ Chronic kidney disease, Males N = 260, Females N = 265.

P-values were based on t-tests. Bold text indicates significant associations.
