# Supplementary material for: Sex differences in biological aging and the association with clinical measures in older adults
Source: GeroScience. 2023 Sep 25;46(2):1775–88. doi: 10.1007/s11357-023-00941-z (PMC10828143; doi:10.1007/s11357-023-00941-z)
Supplement: Supplementary file 2 — Supplementary file2 (DOCX 86 KB) [file 11357_2023_941_MOESM2_ESM.docx]

**Supplementary Table 6** DNAm estimated telomere length and DNAm-based components of original GrimAge and GrimAge2 by males and females

|  | **All participants**  **(n = 560)** | **Males**  **(n = 276, 49.3%)** | **Females**  **(n = 284, 50.7%)** |
| --- | --- | --- | --- |
| DNAm estimated telomere length; mean (SD) | 6.63 (0.22) | 6.57 (0.21) | 6.69 (0.22) |
| **DNAm-based components of original GrimAge**  **and GrimAge2** | | | |
| ADM; mean (SD) | 359.83 (20.32) | 347.74 (17.50) | 371.58 (15.40) |
| B2M; mean (SD) | 1809574.00 (100297.70) | 1806813.00 (99740.14) | 1812257.00 (100940.00) |
| Cystatin-C; mean (SD) | 635753.80 (25162.22) | 641487.00 (25417.05) | 630182.10 (23656.69) |
| GDF-15; mean (SD) | 1077.80 (118.06) | 1090.69 (105.65) | 1065.27 (127.93) |
| Leptin; mean (SD) | 10884.62 (3698.84) | 7671.18 (1848.05) | 14007.54 (1961.57) |
| smoking-pack-years; mean (SD) | 14.00 (9.27) | 16.10 (9.87) | 11.95 (8.16) |
| PAI-1; mean (SD) | 19727.07 (2479.57) | 20733.20 (2394.64) | 18749.28 (2150.72) |
| TIMP1; mean (SD) | 36204.11 (923.41) | 36358.33 (906.95) | 36054.24 (916.09) |
| CRP; mean (SD) | 0.60 (0.43) | 0.56 (0.44) | 0.64 (0.41) |
| A1C; mean (SD) | 1.72 (0.03) | 1.72 (0.03) | 1.72 (0.02) |

A1C, log transformed hemoglobin A1C; ADM, adrenomedullin; B2M, beta-2-microglobulin; CRP, log transformed high sensitivity C-reactive protein; GDF-15, growth differentiation factor 15; PAI-1, plasminogen activator inhibitor 1; TIMP-1, tissue inhibitor of metalloproteinases 1.

**Supplementary Table 7** DNAm estimated telomere length according to characteristics of participants

|  | **Males (n = 276)**  **DNAm estimated telomere length**  **Mean (SD)** | **Females (n = 284)**  **DNAm estimated telomere length**  **Mean (SD)** |
| --- | --- | --- |
| **Years of education** |  |  |
| <12 years | 6.59 (0.20) | 6.67 (0.21) |
| ≥12 years | 6.56 (0.22) | 6.70 (0.22) |
| **P-value** | 0.22 | 0.25 |
| **Living situation** |  |  |
| At home alone | 6.56 (0.23) | 6.66 (0.25) |
| With family or others | 6.58 (0.21) | 6.71 (0.19) |
| **P-value** | 0.73 | **0.03** |
| **Socioeconomic status (SES)** |  |  |
| Very low | 6.51 (0.25) | 6.63 (0.25) |
| Low | 6.59 (0.16) | 6.67 (0.26) |
| Middle | 6.57 (0.20) | 6.68 (0.20) |
| High | 6.58 (0.19) | 6.68 (0.20) |
| Very high | 6.58 (0.23) | 6.71 (0.22) |
| **P-value** | 0.66 | 0.44 |
| **Smoking** |  |  |
| Never | 6.58 (0.23) | 6.70 (0.22) |
| Former | 6.57 (0.20) | 6.67 (0.21) |
| Current | 6.57 (0.20) | 6.66 (0.18) |
| **P-value** | 0.91 | 0.44 |
| **Alcohol consumption** |  |  |
| Never | 6.59 (0.15) | 6.65 (0.24) |
| Former | 6.59 (0.29) | 6.71 (0.23) |
| Current-Low Risk | 6.57 (0.22) | 6.71 (0.21) |
| Current-High Risk | 6.57 (0.21) | 6.66 (0.19) |
| **P-value** | 0.95 | 0.29 |

P-values were based on t-tests or one-way ANOVA tests, as appropriate. Bold text indicates significant associations.

**Supplementary Table 8** Pearson's correlation matrix for DNAm estimated telomere length and clinical measures (systolic blood pressure (SBP), diastolic blood pressure (DBP), grip strength, gait speed, self-rated health (higher scores indicating worse health), and physical component score (PCS), and mental component score (MCS) of the SF-12), separately in males and females

|  | **SBP** | **DBP** | **Grip Strength** | **Gait Speed** | **Self-Reported Health** | **PCS** | **MCS** |
| --- | --- | --- | --- | --- | --- | --- | --- |
| **Males** | | | | | | | |
| DNAm estimated telomere length | -0.02  0.68 | 0.09  0.12 | 0.08  0.20 | 0.08  0.16 | 0.01  0.87 | -0.01  0.92 | 0.01  0.83 |
| **Females** | | | | | | | |
| DNAm estimated telomere length | -0.19  **0.001** | -0.10  0.09 | 0.23  **<0.001** | 0.19  **0.001** | -0.14  **0.02** | 0.21  **<0.001** | -0.05  0.42 |

Bold text indicates significant correlations.

**Supplementary Table 9** DNAm estimated telomere length according to chronic conditions

|  | **Males (n = 276)**  **DNAm estimated telomere length**  **Mean (SD)** | **Females (n = 284)**  **DNAm estimated telomere length**  **Mean (SD)** |
| --- | --- | --- |
| **Hypertension** |  |  |
| Yes | 6.57 (0.21) | 6.68 (0.22) |
| No | 6.57 (0.22) | 6.71 (0.22) |
| **P-value** | 0.98 | 0.27 |
| **Diabetes** |  |  |
| Yes | 6.53 (0.26) | 6.67 (0.18) |
| No | 6.58 (0.20) | 6.69 (0.22) |
| **P-value** | 0.11 | 0.73 |
| **Dyslipidemia** |  |  |
| Yes | 6.58 (0.22) | 6.69 (0.22) |
| No | 6.57 (0.22) | 6.68 (0.19) |
| **P-value** | 0.62 | 0.77 |
| **Obesity ^a^** |  |  |
| Yes | 6.59 (0.21) | 6.66 (0.22) |
| No | 6.57 (0.22) | 6.70 (0.21) |
| **P-value** | 0.39 | 0.14 |
| **Chronic Kidney Disease ^b^** |  |  |
| Yes | 6.54 (0.20) | 6.63 (0.22) |
| No | 6.58 (0.22) | 6.70 (0.22) |
| **P-value** | 0.14 | **0.03** |
| **Depression** |  |  |
| Yes | 6.55 (0.22) | 6.68 (0.25) |
| No | 6.58 (0.21) | 6.69 (0.21) |
| **P-value** | 0.53 | 0.85 |

^a^ Obesity, Males N = 274, Females N = 283;

^b^ Chronic kidney disease, Males N = 260, Females N = 265.

P-values were based on t-tests. Bold text indicates significant associations.

**Supplementary Table 10** DNAm-based components of original GrimAge and GrimAge2 according to characteristics of males in the study (n = 276)

|  | **ADM** | **B2M** | **Cystatin-C** | **GDF-15** | **Leptin** | **smoking-pack-years** | **PAI-1** | **TIMP1** | **CRP** | **A1C** |
| --- | --- | --- | --- | --- | --- | --- | --- | --- | --- | --- |
| **Years of education** |  |  |  |  |  |  |  |  |  |  |
| <12 years | 347.64  (16.01) | 1804700.00  (98340.48) | 640186.10  (22782.73) | 1087.40  (106.11) | 7509.62  (1703.10) | 16.43  (9.86) | 20734.34  (2438.08) | 36387.66  (837.71) | 0.55  (0.41) | 1.71  (0.02) |
| ≥12 years | 347.80  (18.48) | 1808234.00  (100943.80) | 642362.20  (27077.93) | 1092.90  (105.60) | 7779.86  (1936.96) | 15.87  (9.91) | 20732.43  (2372.44) | 36338.60  (952.69) | 0.56  (0.46) | 1.72  (0.03) |
| **P-value** | 0.94 | 0.77 | 0.49 | 0.67 | 0.23 | 0.64 | 0.99 | 0.66 | 0.78 | 0.36 |
| **Living situation** |  |  |  |  |  |  |  |  |  |  |
| At home alone | 350.09  (21.07) | 1838369.00 (106555.40) | 647444.30  (25127.52) | 1108.74  (91.10) | 8067.01  (2092.20) | 16.91  (10.73) | 20948.27  (2887.69) | 36498.13  (917.06) | 0.53  (0.46) | 1.72  (0.03) |
| With family or others | 347.26  (16.68) | 1800336.00 (97265.91) | 640264.40  (25357.35) | 1086.99  (108.20) | 7589.94  (1788.09) | 15.93  (9.71) | 20689.05  (2285.28) | 36329.64  (904.20) | 0.56  (0.44) | 1.72  (0.03) |
| **P-value** | 0.31 | **0.02** | 0.08 | 0.20 | 0.11 | 0.54 | 0.50 | 0.25 | 0.64 | 0.44 |
| **Socioeconomic status (SES)** |  |  |  |  |  |  |  |  |  |  |
| Very low | 347.34  (13.06) | 1789397.80  (81601.08) | 639059.43  (22348.08) | 1075.50  (105.53) | 7929.28  (1462.26) | 16.81  (5.87) | 22096.71  (2394.28) | 36360.98  (705.93) | 0.54 (0.45) | 1.74  (0.02) |
| Low | 344.15  (18.52) | 1799375.10  (101710.51) | 633730.45  (30145.36) | 1069.64  (104.62) | 7971.16  (1520.70) | 16.53  (7.97) | 20344.55  (2395.33) | 36284.78  (874.93) | 0.37  (0.40) | 1.72  (0.03) |
| Middle | 345.62  (20.60) | 1826486.40  (102513.79) | 640824.89  (23142.61) | 1094.41  (103.42) | 7920.60  (1936.93) | 15.85  (8.89) | 20748.21  (2165.03) | 36449.68  (994.98) | 0.57  (0.48) | 1.72  (0.03) |
| High | 348.40  (16.57) | 1812319.80  (105562.06) | 643069.83  (31318.10) | 1083.44  (115.75) | 7569.80  (2054.53) | 17.78  (12.03) | 20517.71  (2518.18) | 36353.34  (1008.98) | 0.59  (0.52) | 1.72  (0.03) |
| Very high | 348.69  (17.53) | 1801595.40  (99291.29) | 642327.90  (22830.27) | 1096.89  (100.70) | 7558.57  (1830.30) | 15.16  (9.92) | 20669.65  (2355.92) | 36337.02  (875.29) | 0.57  (0.40) | 1.71  (0.03) |
| **P-value** | 0.76 | 0.56 | 0.67 | 0.73 | 0.67 | 0.54 | 0.07 | 0.96 | 0.44 | **0.002** |
| **Smoking** |  |  |  |  |  |  |  |  |  |  |
| Never | 346.96  (16.04) | 1804012.70 (95446.73) | 638789.89  (22483.77) | 1083.39 (100.02) | 7377.29 (1782.73) | 11.97  (5.21) | 20381.38  (2277.84) | 36260.16  (841.13) | 0.51 (0.41) | 1.71  (0.02) |
| Former | 347.76  (18.65) | 1803101.20 (100239.75) | 643002.28  (28034.15) | 1090.82 (104.03) | 7962.32 (1936.03) | 17.89  (9.41) | 21071.92  (2452.31) | 36420.48  (956.89) | 0.60  (0.47) | 1.72  (0.03) |
| Current | 355.98  (18.43) | 1878668.40 (119989.88) | 653434.70  (20619.78) | 1167.66 (153.98) | 7555.05 (820.18) | 40.31  (12.83) | 20704.58  (2619.57) | 36714.47  (937.20) | 0.66  (0.39) | 1.71  (0.03) |
| **P-value** | 0.23 | **0.04** | 0.10 | **0.03** | **0.04** | **<0.001** | 0.06 | 0.14 | 0.18 | 0.24 |
| **Alcohol consumption** |  |  |  |  |  |  |  |  |  |  |
| Never | 349.21  (13.95) | 1835267.20  (109480.67) | 641161.02  (26309.70) | 1089.36  (115.46) | 6865.18  (1568.21) | 16.03  (8.41) | 20498.04  (2151.06) | 36544.50  (868.74) | 0.58  (0.45) | 1.72  (0.02) |
| Former | 344.00  (10.86) | 1785725.10  (87160.24) | 644707.52  (19885.15) | 1095.90  (75.51) | 7861.92  (2010.16) | 15.98  (9.86) | 21273.00  (2775.04) | 36515.20  (798.55) | 0.49  (0.34) | 1.72  (0.03) |
| Current-Low Risk | 348.39  (17.11) | 1802869.30  (97768.04) | 640877.00  (26041.36) | 1089.07  (107.83) | 7732.35  (1973.79) | 15.00  (9.82) | 20527.42  (2330.02) | 36288.29  (919.32) | 0.55  (0.43) | 1.72  (0.03) |
| Current-High Risk | 346.57  (19.95) | 1808846.90  (102190.22) | 642300.47  (24996.35) | 1093.48  (103.68) | 7772.37  (1604.62) | 18.27  (10.21) | 21127.20  (2511.67) | 36414.26  (909.64) | 0.58  (0.48) | 1.71  (0.03) |
| **P-value** | 0.73 | 0.42 | 0.95 | 0.99 | 0.15 | 0.12 | 0.24 | 0.45 | 0.87 | 0.84 |

A1C, log transformed hemoglobin A1C; ADM, adrenomedullin; B2M, beta-2-microglobulin; CRP, log transformed high sensitivity C-reactive protein; GDF-15, growth differentiation factor 15; PAI-1, plasminogen activator inhibitor 1; TIMP-1, tissue inhibitor of metalloproteinases 1.

P-values were based on t-tests or one-way ANOVA tests, as appropriate. Bold text indicates significant associations.

**Supplementary Table 11** DNAm-based components of original GrimAge and GrimAge2 according to characteristics of females in the study (n = 284)

|  | **ADM** | **B2M** | **Cystatin-C** | **GDF-15** | **Leptin** | **smoking-pack-years** | **PAI-1** | **TIMP1** | **CRP** | **A1C** |
| --- | --- | --- | --- | --- | --- | --- | --- | --- | --- | --- |
| **Years of education** |  |  |  |  |  |  |  |  |  |  |
| <12 years | 373.57  (15.00) | 1816635.00  (101340.00) | 630571.60  (23742.71) | 1061.04  (107.62) | 14229.61  (1948.88) | 12.19  (8.26) | 19009.48  (2117.03) | 36097.51  (930.83) | 0.69  (0.43) | 1.72  (0.03) |
| ≥12 years | 370.06  (15.57) | 1808913.00  (100821.00) | 629884.40  (23660.55) | 1068.51  (141.74) | 13837.89  (1960.30) | 11.77  (8.10) | 18550.49  (2161.55) | 36021.17  (906.19) | 0.60  (0.39) | 1.71  (0.02) |
| **P-value** | 0.06 | 0.52 | 0.81 | 0.63 | 0.10 | 0.67 | 0.07 | 0.49 | 0.09 | **0.004** |
| **Living situation** |  |  |  |  |  |  |  |  |  |  |
| At home alone | 374.23 (16.61) | 1837595.00 (106434.20) | 632907.70  (25423.12) | 1061.63  (105.48) | 14049.06  (2046.00) | 12.14  (7.73) | 18549.12  (2183.55) | 36165.65  (1033.94) | 0.61 (0.41) | 1.72  (0.03) |
| With family or others | 369.84 (14.34) | 1795758.00 (93886.05) | 628407.20  (22329.37) | 1067.64  (140.88) | 13980.51  (1910.18) | 11.83  (8.44) | 18879.62  (2125.30) | 35981.68  (825.55) | 0.66  (0.41) | 1.72  (0.02) |
| **P-value** | **0.02** | **<0.001** | 0.12 | 0.70 | 0.77 | 0.76 | 0.21 | 0.10 | 0.30 | 0.89 |
| **Socioeconomic status (SES)** |  |  |  |  |  |  |  |  |  |  |
| Very low | 374.33  (13.56) | 1822677.70  (83055.63) | 631668.73  (24490.98) | 1055.90  (98.86) | 14369.64  (2268.68) | 10.90  (5.65) | 19486.98  (1858.62) | 36281.81  (877.74) | 0.88 (0.49) | 1.73  (0.03) |
| Low | 372.95  (17.55) | 1842481.80  (130925.12) | 630843.95  (25349.80) | 1070.91  (145.84) | 14466.03  (2220.00) | 13.54  (12.67) | 19101.30  (2289.80) | 36241.34  (913.89) | 0.59  (0.46) | 1.73  (0.02) |
| Middle | 371.22  (16.47) | 1805415.00  (90242.45) | 632830.76  (23978.07) | 1101.42  (192.86) | 13676.42  (1849.69) | 14.88  (9.47) | 18904.69  (2608.62) | 36094.61  (814.87) | 0.61  (0.38) | 1.72  (0.03) |
| High | 372.70  (14.30) | 1824679.70  (101868.32) | 630581.86  (20673.70) | 1058.37  (93.48) | 13995.58  (2140.68) | 13.14  (9.37) | 18902.48  (1886.78) | 35983.08  (998.80) | 0.63  (0.44) | 1.72  (0.02) |
| Very high | 370.39  (15.53) | 1801089.60  (99889.96) | 628750.65  (24577.23) | 1057.62  (116.48) | 13962.93  (1797.06) | 10.39  (5.86) | 18423.81  (2100.80) | 35995.19  (915.85) | 0.62  (0.37) | 1.71  (0.02) |
| **P-value** | 0.72 | 0.27 | 0.89 | 0.38 | 0.48 | **0.01** | 0.13 | 0.47 | 0.05 | **0.001** |
| **Smoking** |  |  |  |  |  |  |  |  |  |  |
| Never | 371.67  (15.07) | 1817865.70  (107591.04) | 630787.42  (24474.46) | 1063.83  (135.15) | 14009.61  (1956.72) | 8.83  (4.98) | 18526.62 (2033.65) | 36104.77  (893.13) | 0.61 (0.40) | 1.72  (0.02) |
| Former | 370.98  (16.31) | 1801151.50  (85000.65) | 627981.35  (21802.62) | 1058.27  (112.34) | 13958.13  (1963.16) | 15.98  (7.85) | 19044.61 (2330.71) | 35949.10  (968.02) | 0.67  (0.43) | 1.72  (0.03) |
| Current | 375.78  (12.99) | 1811740.50  (112950.14) | 640369.35  (24124.96) | 1166.14  (88.06) | 14470.70  (2213.93) | 34.33  (10.74) | 20257.83 (1747.38) | 36101.53  (832.57) | 0.81  (0.38) | 1.73  (0.03) |
| **P-value** | 0.67 | 0.43 | 0.28 | 0.05 | 0.76 | **<0.001** | **0.02** | 0.41 | 0.23 | **0.04** |
| **Alcohol consumption** |  |  |  |  |  |  |  |  |  |  |
| Never | 372.43  (15.41) | 1824213.90  (110359.32) | 632766.42  (24404.31) | 1085.33  (172.99) | 13851.23  (1655.94) | 11.60  (7.54) | 18140.40  (1860.72) | 36200.46  (978.87) | 0.59 (0.42) | 1.72 (0.02) |
| Former | 369.54  (14.31) | 1821355.80  (62210.17) | 638143.01  (23833.32) | 1149.70  (201.67) | 12979.62  (1685.50) | 24.49  (14.11) | 19191.32  (2866.19) | 36148.85  (604.16) | 0.60  (0.49) | 1.73  (0.03) |
| Current-Low Risk | 371.77  (15.53) | 1808159.30  (100808.79) | 629473.65  (24270.27) | 1057.18  (110.96) | 14076.89  (2033.20) | 11.47  (7.97) | 18878.32  (2125.06) | 35993.12  (920.67) | 0.64  (0.38) | 1.72  (0.02) |
| Current-High Risk | 370.05  (15.43) | 1812016.70  (96836.65) | 628094.20  (19878.62) | 1055.24  (99.82) | 14138.62  (2059.97) | 11.79  (5.95) | 18905.02  (2373.19) | 36101.49  (866.60) | 0.72  (0.50) | 1.71  (0.03) |
| **P-value** | 0.86 | 0.77 | 0.54 | 0.10 | 0.36 | **<0.001** | 0.13 | 0.50 | 0.49 | 0.11 |

A1C, log transformed hemoglobin A1C; ADM, adrenomedullin; B2M, beta-2-microglobulin; CRP, log transformed high sensitivity C-reactive protein; GDF-15, growth differentiation factor 15; PAI-1, plasminogen activator inhibitor 1; TIMP-1, tissue inhibitor of metalloproteinases 1.

P-values were based on t-tests or one-way ANOVA tests, as appropriate. Bold text indicates significant associations.

**Supplementary Table 12** Pearson's correlation matrix for DNAm-based components of original GrimAge and GrimAge2 and clinical measures (systolic blood pressure (SBP), diastolic blood pressure (DBP), grip strength, gait speed, self-rated health (higher scores indicating worse health), and physical component score (PCS), and mental component score (MCS) of the SF-12) in males (n = 276)

|  | **ADM** | **B2M** | **Cystatin-C** | **GDF-15** | **Leptin** | **smoking-pack-years** | **PAI-1** | **TIMP1** | **CRP** | **A1C** |
| --- | --- | --- | --- | --- | --- | --- | --- | --- | --- | --- |
| **SBP** | 0.08  0.19 | 0.06  0.29 | 0.05  0.38 | -0.01  0.86 | -0.18  **0.003** | 0.09  0.16 | 0.00  0.96 | 0.07  0.25 | -0.02  0.76 | 0.02  0.71 |
| **DBP** | -0.08  0.18 | -0.15  **0.01** | -0.20  **<0.001** | -0.15  **0.01** | -0.16  **0.01** | -0.09  0.14 | -0.07  0.24 | -0.23  **<0.001** | -0.08  0.20 | -0.03  0.59 |
| **Grip Strength** | -0.18  **0.003** | -0.21  **<0.001** | -0.25  **<0.001** | -0.09  0.13 | 0.03  0.60 | -0.11  0.07 | -0.04  0.51 | -0.36  **<0.001** | -0.09  0.13 | -0.03  0.60 |
| **Gait Speed** | -0.08  0.21 | -0.12  0.05 | -0.21  **<0.001** | -0.10  0.11 | 0.02  0.79 | -0.17  **0.005** | -0.07  0.23 | -0.19  **0.002** | -0.12  **0.04** | 0.00  0.98 |
| **Self-Rated Health** | -0.02  0.75 | 0.01  0.92 | 0.06  0.31 | 0.02  0.73 | -0.00  0.97 | 0.01  0.86 | 0.16  **0.01** | 0.03  0.63 | 0.06  0.32 | 0.12  **0.04** |
| **PCS** | -0.05  0.40 | -0.06  0.28 | -0.14  **0.02** | -0.00  0.94 | -0.05  0.44 | -0.14  **0.02** | -0.18  **0.002** | -0.19  **0.002** | -0.09  0.14 | -0.15  **0.01** |
| **MCS** | -0.02  0.70 | 0.00  0.98 | 0.00  0.97 | -0.01  0.82 | 0.01  0.86 | 0.11  0.07 | 0.05  0.42 | 0.06  0.29 | 0.10  0.08 | 0.11  0.07 |

A1C, log transformed hemoglobin A1C; ADM, adrenomedullin; B2M, beta-2-microglobulin; CRP, log transformed high sensitivity C-reactive protein; GDF-15, growth differentiation factor 15; PAI-1, plasminogen activator inhibitor 1; TIMP-1, tissue inhibitor of metalloproteinases 1.

Bold text indicates significant correlations.

**Supplementary Table 13** Pearson's correlation matrix for DNAm-based components of original GrimAge and GrimAge2 and clinical measures (systolic blood pressure (SBP), diastolic blood pressure (DBP), grip strength, gait speed, self-rated health (higher scores indicating worse health), and physical component score (PCS), and mental component score (MCS) of the SF-12) in females (n = 284)

|  | **ADM** | **B2M** | **Cystatin-C** | **GDF-15** | **Leptin** | **smoking-pack-years** | **PAI-1** | **TIMP1** | **CRP** | **A1C** |
| --- | --- | --- | --- | --- | --- | --- | --- | --- | --- | --- |
| **SBP** | 0.06  0.28 | 0.14  **0.02** | 0.13  **0.03** | 0.11  0.07 | 0.07  0.23 | 0.09  0.13 | 0.12  0.05 | 0.13  **0.02** | 0.12  0.05 | 0.22  **<0.001** |
| **DBP** | -0.03  0.64 | -0.06  0.35 | -0.10  0.10 | -0.09  0.12 | -0.04  0.52 | 0.03  0.65 | -0.03  0.67 | -0.02  0.71 | 0.05  0.38 | 0.01  0.80 |
| **Grip Strength** | -0.15  **0.01** | -0.34  **<0.001** | -0.27  **<0.001** | -0.11  0.07 | -0.04  0.56 | -0.01  0.91 | 0.02  0.76 | -0.35  **<0.001** | -0.01  0.85 | -0.10  0.09 |
| **Gait Speed** | -0.16  **0.01** | -0.21  **<0.001** | -0.11  0.06 | -0.11  0.06 | -0.14  **0.02** | -0.06  0.29 | -0.08  0.17 | -0.28  **<0.001** | -0.05  0.39 | -0.16  **0.01** |
| **Self-Rated Health** | 0.06  0.31 | 0.10  0.08 | 0.09  0.15 | 0.15  **0.01** | 0.07  0.24 | 0.09  0.13 | 0.14  **0.02** | 0.07  0.27 | 0.19  **0.002** | 0.13  **0.03** |
| **PCS** | -0.16  **0.01** | -0.16  **0.01** | -0.13  **0.03** | -0.13  **0.03** | -0.06  0.32 | -0.11  0.06 | -0.13  **0.03** | -0.17  **0.004** | -0.20  **<0.001** | -0.13  **0.03** |
| **MCS** | -0.04  0.55 | -0.02  0.76 | 0.01  0.86 | 0.05  0.39 | -0.12  0.05 | -0.06  0.28 | -0.01  0.85 | 0.05  0.36 | -0.07  0.25 | -0.01  0.82 |

A1C, log transformed hemoglobin A1C; ADM, adrenomedullin; B2M, beta-2-microglobulin; CRP, log transformed high sensitivity C-reactive protein; GDF-15, growth differentiation factor 15; PAI-1, plasminogen activator inhibitor 1; TIMP-1, tissue inhibitor of metalloproteinases 1.

Bold text indicates significant correlations.

**Supplementary Table 14** DNAm-based components of original GrimAge and GrimAge2 according to chronic conditions (Males, n = 276)

|  | **ADM** | **B2M** | **Cystatin-C** | **GDF-15** | **Leptin** | **smoking-pack-years** | **PAI-1** | **TIMP1** | **CRP** | **A1C** |
| --- | --- | --- | --- | --- | --- | --- | --- | --- | --- | --- |
| **Hypertension** |  |  |  |  |  |  |  |  |  |  |
| Yes | 348.92  (16.30) | 1807176.00  (95522.11) | 641741.40  (24033.29) | 1088.00  (104.48) | 7538.24  (1752.00) | 16.35  (9.66) | 20774.16  (2522.56) | 36379.90  (829.09) | 0.58  (0.40) | 1.72  (0.03) |
| No | 344.80  (19.99) | 1805906.00  (110200.20) | 640852.60  (28734.70) | 1097.39  (108.89) | 8002.69  (2042.55) | 15.46  (10.44) | 20631.04  (2052.43) | 36304.53  (1081.05) | 0.50  (0.54) | 1.71  (0.03) |
| **P-value** | 0.08 | 0.92 | 0.79 | 0.51 | 0.06 | 0.50 | 0.65 | 0.53 | 0.15 | 0.16 |
| **Diabetes** |  |  |  |  |  |  |  |  |  |  |
| Yes | 352.25  (19.21) | 1813565.00  (101583.20) | 646299.30  (25997.62) | 1128.12  (126.43) | 7971.80  (1862.85) | 17.61  (10.82) | 22030.43  (2259.08) | 36421.57  (817.99) | 0.74  (0.36) | 1.73  (0.02) |
| No | 346.91  (17.08) | 1805567.00  (99568.03) | 640598.90  (25265.05) | 1083.78  (100.15) | 7615.70  (1843.97) | 15.82  (9.69) | 20493.79  (2346.01) | 36346.66  (923.58) | 0.52  (0.45) | 1.71  (0.03) |
| **P-value** | 0.07 | 0.63 | 0.18 | **0.01** | 0.25 | 0.28 | **<0.001** | 0.62 | **0.003** | **<0.001** |
| **Dyslipidemia** |  |  |  |  |  |  |  |  |  |  |
| Yes | 347.48  (15.59) | 1793294.00  (95902.85) | 639306.5  (24875.81) | 1079.89  (108.45) | 7620.89  (1768.70) | 16.51  (9.97) | 20961.96  (2474.60) | 36321.97  (806.87) | 0.57  (0.44) | 1.72  (0.03) |
| No | 348.00  (19.33) | 1820729.00 (102030.20) | 643731.7  (25861.79) | 1101.81  (101.89) | 7722.95  (1931.51) | 15.67  (9.79) | 20497.70  (2294.73) | 36395.76  (1001.23) | 0.54  (0.44) | 1.72  (0.03) |
| **P-value** | 0.81 | **0.02** | 0.15 | 0.08 | 0.65 | 0.48 | 0.11 | 0.50 | 0.55 | 0.55 |
| **Obesity ^a^** |  |  |  |  |  |  |  |  |  |  |
| Yes | 344.60  (18.39) | 1776402.00  (80625.46) | 635656.50 (20852.57) | 1077.54  (89.20) | 7756.33  (1491.98) | 16.17  (7.71) | 22045.86  (2269.21) | 36169.48  (740.91) | 0.63 (0.44) | 1.73  (0.03) |
| No | 348.71  (17.14) | 1817060.00 (104058.30) | 643510.00 (26647.59) | 1094.74  (110.79) | 7646.34  (1965.85) | 16.04  (10.57) | 20256.43  (2262.03) | 36422.95  (950.88) | 0.53  (0.44) | 1.71  (0.03) |
| **P-value** | 0.09 | **0.003** | **0.03** | 0.24 | 0.67 | 0.92 | **<0.001** | **0.04** | 0.09 | **<0.001** |
| **Chronic Kidney Disease ^b^** |  |  |  |  |  |  |  |  |  |  |
| Yes | 352.27 (16.16) | 1841003.00 (100298.50) | 651626.10 (31997.97) | 1136.87  (101.24) | 7631.06  (1732.98) | 20.30  (11.69) | 21106.49  (2616.95) | 36735.74  (1004.23) | 0.77  (0.52) | 1.73  (0.04) |
| No | 346.60 (17.84) | 1798673.00  (97894.08) | 638644.30 (22481.17) | 1079.03  (100.39) | 7611.45  (1818.67) | 14.90  (8.49) | 20680.66  (2344.79) | 36264.11  (859.19) | 0.50  (0.41) | 1.71  (0.03) |
| **P-value** | **0.04** | **0.01** | **0.001** | **<0.001** | 0.94 | **<0.001** | 0.25 | **<0.001** | **<0.001** | **0.01** |
| **Depression** |  |  |  |  |  |  |  |  |  |  |
| Yes | 352.19  (18.49) | 1832219.00  (96314.66) | 649331.30  (20668.28) | 1114.59  (106.32) | 8005.18  (1652.11) | 17.04  (9.37) | 20584.43  (2736.49) | 36629.87  (799.66) | 0.58  (0.40) | 1.71  (0.03) |
| No | 347.28  (17.37) | 1804171.00  (99905.30) | 640671.20  (25758.50) | 1088.21  (105.48) | 7636.44  (1866.82) | 16.00  (9.94) | 20748.67  (2361.92) | 36330.09  (914.19) | 0.55  (0.45) | 1.72  (0.03) |
| **P-value** | 0.17 | 0.17 | 0.10 | 0.23 | 0.33 | 0.61 | 0.74 | 0.11 | 0.76 | 0.72 |

A1C, log transformed hemoglobin A1C; ADM, adrenomedullin; B2M, beta-2-microglobulin; CRP, log transformed high sensitivity C-reactive protein; GDF-15, growth differentiation factor 15; PAI-1, plasminogen activator inhibitor 1; TIMP-1, tissue inhibitor of metalloproteinases 1.

^a^ Obesity, Males N = 274

^b^ Chronic kidney disease, Males N = 260

P-values were based on t-tests. Bold text indicates significant associations.

**Supplementary Table 15** DNAm-based components of original GrimAge and GrimAge2 according to chronic conditions (Females, n = 284)

|  | **ADM** | **B2M** | **Cystatin-C** | **GDF-15** | **Leptin** | **smoking-pack-years** | **PAI-1** | **TIMP1** | **CRP** | **A1C** |
| --- | --- | --- | --- | --- | --- | --- | --- | --- | --- | --- |
| **Hypertension** |  |  |  |  |  |  |  |  |  |  |
| Yes | 372.40  (15.15) | 1820657.00  (101850.00) | 632147.70  (24207.64) | 1070.13  (141.60) | 14119.44  (1968.57) | 11.99  (8.17) | 19022.91  (2135.47) | 36132.15  (932.92) | 0.66  (0.42) | 1.72  (0.03) |
| No | 370.06  (15.81) | 1796802.00  (97868.55) | 626565.30  (22274.44) | 1056.34  (97.93) | 13801.66  (1941.57) | 11.89  (8.18) | 18245.8  (2097.11) | 35910.88  (870.71) | 0.60  (0.38) | 1.71  (0.02) |
| **P-value** | 0.22 | 0.06 | 0.06 | 0.39 | 0.19 | 0.92 | **0.004** | 0.05 | 0.22 | **0.001** |
| **Diabetes** |  |  |  |  |  |  |  |  |  |  |
| Yes | 369.42  (14.44) | 1807077.00  (78013.00) | 641326.10 (24536.52) | 1113.13  (130.68) | 14280.74  (2194.45) | 12.81  (6.79) | 19966.69  (2618.04) | 36020.16  (1337.00) | 0.71  (0.47) | 1.73  (0.03) |
| No | 371.76  (15.49) | 1812692.00  (102740.40) | 629246.30 (23388.55) | 1061.26  (127.13) | 13984.60  (1943.66) | 11.88  (8.27) | 18647.05  (2080.52) | 36057.10  (875.23) | 0.63  (0.41) | 1.72  (0.02) |
| **P-value** | 0.50 | 0.80 | **0.02** | 0.07 | 0.50 | 0.61 | **0.01** | 0.86 | 0.41 | **<0.001** |
| **Dyslipidemia** |  |  |  |  |  |  |  |  |  |  |
| Yes | 371.13  (15.98) | 1812133.00  (102008.80) | 629089.40  (23551.13) | 1061.92  (136.07) | 14058.85  (2037.23) | 11.71  (8.27) | 18721.22  (2169.84) | 36010.35  (957.81) | 0.61  (0.40) | 1.72  (0.03) |
| No | 372.86  (13.59) | 1812616.00  (98476.27) | 633340.50  (23839.72) | 1074.98  (100.97) | 13859.25  (1728.81) | 12.66  (7.83) | 18830.37  (2107.10) | 36181.09  (775.17) | 0.73  (0.42) | 1.72  (0.02) |
| **P-value** | 0.41 | 0.97 | 0.19 | 0.45 | 0.45 | 0.39 | 0.71 | 0.17 | **0.03** | 0.35 |
| **Obesity ^a^** |  |  |  |  |  |  |  |  |  |  |
| Yes | 372.33  (12.81) | 1817085.00  (90378.35) | 629203.10  (20286.14) | 1074.31  (113.41) | 14617.72  (1812.70) | 12.24  (7.53) | 19393.13  (2087.17) | 36108.39  (824.74) | 0.76  (0.40) | 1.73  (0.03) |
| No | 371.35  (16.34) | 1811153.00  (104611.90) | 630833.50  (24692.98) | 1062.28  (133.41) | 13765.49  (1974.68) | 11.87  (8.42) | 18511.09  (2115.08) | 36034.56  (953.13) | 0.60  (0.40) | 1.71  (0.02) |
| **P-value** | 0.63 | 0.65 | 0.60 | 0.48 | **<0.001** | 0.73 | **0.002** | 0.54 | **0.003** | **<0.001** |
| **Chronic Kidney Disease ^b^** |  |  |  |  |  |  |  |  |  |  |
| Yes | 374.71  (16.20) | 1844405.00 (103639.60) | 638560.20 (26477.03) | 1087.29  (102.97) | 13892.71  (2380.88) | 11.35  (7.86) | 18958.17  (2294.71) | 36438.11  (1007.44) | 0.66  (0.45) | 1.72  (0.03) |
| No | 370.62  (15.14) | 1804022.00 (100724.00) | 628031.70 (22580.69) | 1060.84  (134.63) | 13983.45  (1857.57) | 11.86  (7.97) | 18636.90  (2122.22) | 35959.27  (871.09) | 0.62  (0.40) | 1.72  (0.02) |
| **P-value** | 0.08 | **0.01** | **0.004** | 0.18 | 0.76 | 0.67 | 0.33 | **<0.001** | 0.55 | 0.19 |
| **Depression** |  |  |  |  |  |  |  |  |  |  |
| Yes | 373.19  (14.11) | 1825828.00  (106317.60) | 634681.40  (23898.03) | 1073.66  (107.47) | 14098.73  (1446.96) | 16.83  (13.47) | 18973.91  (1858.82) | 35928.64  (1230.20) | 0.72  (0.36) | 1.72  (0.02) |
| No | 371.38  (15.56) | 1810594.00  (100355.40) | 629630.80  (23615.63) | 1064.25  (130.36) | 13996.37  (2017.60) | 11.36  (7.06) | 18721.75  (2185.47) | 36069.62  (871.85) | 0.63  (0.42) | 1.72  (0.03) |
| **P-value** | 0.54 | 0.43 | 0.26 | 0.70 | 0.78 | **<0.001** | 0.54 | 0.42 | 0.23 | 0.25 |

A1C, log transformed hemoglobin A1C; ADM, adrenomedullin; B2M, beta-2-microglobulin; CRP, log transformed high sensitivity C-reactive protein; GDF-15, growth differentiation factor 15; PAI-1, plasminogen activator inhibitor 1; TIMP-1, tissue inhibitor of metalloproteinases 1.

^a^ Obesity, Females N = 283;

^b^ Chronic kidney disease, Females N = 265.

P-values were based on t-tests. Bold text indicates significant associations.
